# Benzyl butyl phthalate induces migration, invasion, and angiogenesis of Huh7 hepatocellular carcinoma cells through nongenomic AhR/G-protein signaling

**DOI:** 10.1186/1471-2407-14-556

**Published:** 2014-08-01

**Authors:** Cheng-Fang Tsai, Tsung-Hua Hsieh, Jau-Nan Lee, Chia-Yi Hsu, Yu-Chih Wang, Feng-Jie Lai, Kung-Kai Kuo, Hua-Lin Wu, Eing-Mei Tsai, Po-Lin Kuo

**Affiliations:** Graduate Institute of Medicine, College of Medicine, Kaohsiung Medical University, Kaohsiung City, 807 Taiwan; Department of Obstetrics and Gynecology Kaohsiung Medical University Hospital, Kaohsiung Medical University, No. 100, Zihyou 1st Rd., Sanmin District, Kaohsiung City, 807 Taiwan; Department of Dermatology, Chi-Mei Medical Center, 901 Chung Hwa Road, Yung Kang City, Tainan 701 Taiwan; Division of Hepatobiliary Pancreatic Surgery, Department of Surgery, Kaohsiung Medical University Hospital, Kaohsiung, Taiwan; Department of Biochemistry and Molecular Biology, National Cheng Kung University, Tainan, Taiwan; Cardiovascular Research Center, College of Medicine, National, Cheng Kung University, Tainan, Taiwan; Center for Bioscience and Biotechnology, National Cheng Kung University, Tainan, Taiwan; Center for Resources, Research and Development, Kaohsiung Medical University, Kaohsiung City, 807 Taiwan; Institute of Clinical Medicine, College of Medicine, Kaohsiung Medical University, No. 100, Zihyou 1st Rd., Sanmin District, Kaohsiung Taiwan

**Keywords:** Phthalate, Aryl hydrocarbon receptor, Angiogenesis, Migration, Hepatocellular carcinoma

## Abstract

**Background:**

The widespread use of phthalates as plasticizers has raised public health concerns regarding their adverse effects, including an association with cancer. Although animal investigations have suggested an association between phthalate exposure and hepatocellular carcinoma, the mechanisms are unknown.

**Methods:**

The hepatocellular carcinoma cell line Huh7 was treated with benzyl butyl phthalate (BBP), and then analyzed by total internal reflection fluorescence microscopy, confocal microscopy and double immunogold transmission electron microscopy. Following BBP treatment, mRNA levels were measured by RT-PCR, protein levels were measured using western blot, and vascular endothelial growth factor levels were measured by an enzyme-linked immunosorbent assay. Cell migration and invasion assays were evaluated by transwell, and angiogenesis were performed by a tube formation assay. Nude mice were used to investigate metastasis and angiogenesis *in vivo*.

**Results:**

BBP affected hepatocellular carcinoma progression through the aryl hydrocarbon receptor (AhR) and that benzyl butyl phthalate (BBP) stimulated AhR at the cell surface, which then interacted with G proteins and triggered a downstream signaling cascade. BBP activated AhR through a nongenomic action involving G-protein signaling rather than the classical genomic AhR action. BBP treatment promoted cell migration and invasion *in vitro* and metastasis *in vivo* via the AhR/G_β_/PI3K/Akt/NF-κB pathway. In addition, BBP induced both *in vitro* and *in vivo* angiogenesis through the AhR/ERK/VEGF pathway.

**Conclusions:**

These findings suggest a novel nongenomic AhR mechanism involving G-protein signaling induced by phthalates, which contributes to tumor progression of hepatocellular carcinoma.

**Electronic supplementary material:**

The online version of this article (doi:10.1186/1471-2407-14-556) contains supplementary material, which is available to authorized users.

## Background

Globally, hepatocellular carcinoma is the sixth most common cancer and the third most common cause of cancer-related deaths [1]. Risk factors for hepatocellular carcinoma include infection with hepatitis B or C viruses, alcohol consumption, smoking, and environmental factors [2]. Oral administration of phthalates to rats results in liver enlargement and cause increased malondialdehyde levels in the liver, indicating that phthalates cause oxidative damage of the liver [3]. Bis(2-ethylhexyl) phthalate (DEHP) acts as a promoter of hepatocellular tumors initiated by *N*-nitrosodiethylamine [4]. Moreover, lifetime DEHP treatment induces testis and liver cancer in rats [5]. Based on proteomic analysis, the proteins secreted by HepG2 cells that have been treated with benzyl butyl phthalate (BBP) are associated with DNA damage, tumor progression, apoptosis, energy metabolism, and cell structure and motility [6]. The observed roles of BBP in DNA damage and methylation, as well as cell migration, invasion, and proliferation suggest the involvement of BBP in tumor development and progression. These findings support carcinogenesis induced by phthalates, but the mechanism remains largely unknown.

Previous studies have shown that phthalates affect the activation of the aryl hydrocarbon receptor (AhR) [7, 8]. Moreover, phthalates suppressed type I interferon expression in human plasmacytoid dendritic cells via AhR [9]. Our previous study showed that BBP induces necrosis in human granulosa cells via AhR activation followed by downstream CYP1B1 induction [10]. We also showed that phthalates induce proliferation and invasiveness of breast cancer through the AhR/HDAC6/c-Myc signaling pathway [11]. These results suggest that AhR is an important receptor that mediates multiple biological effects of phthalates.

The ligand-activated transcriptional factor AhR regulates the enzymatic functions needed for xenobiotic metabolism. Previous reports revealed two pathways that mediate AhR effects including genomic and non-genomic pathways. The classical genomic function involves AhR nuclear translocation and binding to xenobiotic responsive elements located in the promoters of target genes, CYP1A1 and CYP1B1 [12]. In addition to the classical genomic AhR function, AhR can regulate gene expression through a nongenomic mechanism. The Study of nongenomic signaling is important in the field of toxicology. It is difficult to identify dioxin response element (DRE)-based target genes and many reports suggest that the toxic effect of 2, 3, 7, 8-tetrachlorodibenzo-p-dioxin (TCDD) is more compatible with the nongenomic signaling of AhR, rather than the genomic action [13]. Moreover, our previous study reported a phthalate mediated AhR/HDAC6/c-Myc pathway that demonstrated a nongenomic effect of AhR [10]. Several studies have reported that TCDD induces inflammatory responses through a nongenomic AhR function [14–17], although this nongenomic AhR function remains poorly understood.

Here, we found that BBP promotes angiogenesis, migration and invasion *in vitro* as well as angiogenesis and metastasis *in vivo* of hepatocellular carcinoma. Because G-protein signaling is involved in the regulation of AhR stability [18], we further investigated the AhR function and its possible relationship to G-protein signaling in hepatocellular carcinoma.

Additionally, we revealed that the mechanism through which phthalates activate the nongenomic AhR pathway is associated with G-protein signaling.

## Methods

### Chemicals and plasmid

Fluo-4 was purchased from Invitrogen (Carlsbad, CA, USA). BBP, 2-aminoethoxydiphenyl borate (2-APB), and 6-diamidino-2-phenylindole (DAPI) were purchased from Sigma-Aldrich Co. (St. Louis, MO, USA). Pd98059 and wortmannin were obtained from Calbiochem-Novabiochem (San Diego, CA, USA).

pEGFP-C1-AhR, a kind gift from Dr. Hsin-yu Lee (Department of Life Science, National Taiwan University), was cloned the AhR gene into pEGFP-C1 (Clontech).

### Cell culture

Huh7 cells were cultured in Dulbecco’s modified Eagle’s medium (DMEM) (Life Technologies, Grand Island, NY, USA), PLC/PRF/5 and HepG2 cells were cultured in minimum essential medium (MEM) (Life Technologies, Grand Island, NY, USA) and supplemented with 10% fetal bovine serum (Gibco, California,CA, USA), 1% penicillin (100 U/mL), streptomycin (10 μg/mL), and amphotericin-B (250 μg/mL) (Sigma-Aldrich Co, St. Louis, MO). Human umbilical vein endothelial cells (HUVEC) were grown in EGM-2 medium (Lonza, Basel, Switzerland). All cells were cultured at 37°C in 5% CO_2_.

### Total internal reflection fluorescennce microscopy

For total internal reflection fluorescennce (TIRF) microscopy studies, Huh7 cells were transfected with pEGFP-C1-AhR or pEGFP-C1 as a control using LT1 transfection reagent (Mirus, Madison, WI, USA). After transfection for 24 hours, the cells were harvested and cultured on coverslips for 1 day. Cells were then treated with DMSO as a control or BBP (1 μM) and analyzed by TIRF microscopy (Carl Zeiss, Oberkochen, Germany). GFP intensity was analyzed by Axio Vision Rel. 4.8 software (Carl Zeiss, Oberkochen, Germany).

### Calcium imaging

Calcium imaging was performed using the same method as in a previous study [19] with some modifications. For live cell calcium imaging, Cell-R software was used for microscopy (Olympus, Japan). Huh7 cells were seeded on coverslips and cultured for 24 hours. Cells were incubated with 1 μM Fluo-4, a Ca^2+^-specific dye, at 37°C for 20 minutes in Buffer Salt Saline (BSS) (2 mM CaCl_2_, 5.5 mM d-glucose, 130 mM NaCl, 5.4 mM KCl, 20 mM HEPES pH = 7.4, 1 mM MgSO_4_) and then washed three times before measuring the relative fluorescence intensity. Cells were pretreated with various concentrations of 2-APB for 10 minutes, and then loaded with 1 μM Fluo-4 for 20 minutes. After washing, cells were maintained in calcium-free medium (5.5 mM d-glucose, 130 mM NaCl, 5.4 mM KCl, 20 mM HEPES (pH = 7.4), and 3 mM MgSO_4_) during the experimental periods. The cells were then stimulated by adding BBP (1 μM) after 1 minute. Data were analyzed with Cell-R software.

### Confocal microscopy

Huh7 cells were transfected with pEGFP-C1-AhR using LT1 transfection reagent. After overnight transfection, the cells were harvested and cultured on coverslips for 1 day. BBP (1 μM) was added to stimulate the cells before analysis by confocal microscopy. GFP intensity was analyzed by FV10-ASW 3.0 software (Olympus, Japan).

### Double immunogold transmission electron microscopy

Ultrathin sections of plastic-embedded cells were pretreated with 5% sodium metaperiodate (10 min) by microwave fixation and processing. The grids were incubated with an aliquot of IgG antibodies against AhR or Gα_q/11_ (Santa Cruz Biotechnology, Santa Cruz, CA, USA) followed by probing with secondary antimouse IgG gold particles (6 nm) or anti rabbit IgG gold particles (20 nm), respectively. After washing, the sections were blocked by placing the grids on a drop of phosphate-buffered saline (PBS) containing 1% ovalbumin and incubating for 15 minutes. Sections were then stained with uranyl acetate and lead citrate for characterization by transmission electron microscopy (H-700, Hitachi, Japan).

### Fluorescence in situ hybridization

After treatment with BBP (1 μM) or DMSO for the control group, cells were fixed by adding fixation solution (in 3.7% formaldehyde/PBS) at room temperature for 10 minutes. The cells were washed with PBS twice and then permeabilized by adding 70% EtOH at 4°C for 1 hour. Cells were then washed in wash buffer (5 mL 20× saline-sodium citrate (SSC), 5 mL formamide, and nuclease-free water to a final volume of 50 mL) for 5 minutes. Hybridization was performed by mixing 100 μL of hybridization solution (1 g dextran sulfate, 20 × SSC, 1 mL formamide for a 10% final concentration and nuclease-free water to a final volume of 10 mL) with a specific AhR probe (Stellaris™ FISH Probes, Biosearch Technology, Novato, CA, USA) and incubating the mixture overnight at 37°C. Cells were then stained with DAPI (1 μg/mL) for 5 minutes. After washing, a drop of mounting solution was applied. The slides were then covered with the cell-attached cover glasses and sealed with nail polish. Imaging was performed by confocal microscopy.

### RNA isolation and RT-PCR

Huh7 cells (3 × 10^5^) were seeded in six-well plates, cultured for 24 hours, and then incubated overnight in serum free medium. The cells were then treated with BBP (1 μM) for various times intervals. After stimulation, cells were washed twice with PBS. Total RNA was extracted with TRIzol (Invitrogen). The RNA (2 μg) was applied to a Reverse Transcription System (Promega Biosciences, San Luis Obispo, CA, USA) to synthesize cDNA. The cDNA was then amplified by specific primers. The primer pairs were as follows: AhR, forward 5′-TACTCTGCCGCCCAA ACTGG-3′, reverse 5′-GCTCTGCAACCTCCGATTCC-3′; β-actin, forward 5′-CTCGCTGTCCACCTTCCA-3′, reverse 5′-GCTGTCACCTTCACCGTTC-3′. The PCR conditions were 95°C for 5 min, and then 34 cycles of 95°C for 30 sec, 54°C for 30 sec, and 72°C for 1 min, and a final extension at 72°C for 10 min. PCR products were separated on 2% agarose gels and visualized using ethidium bromide.

### siRNA and shRNA transfection

The following siRNAs were used: scrambled siRNA sense: 5′-GAUCAUACGUGCGAUCAGA-3′, antisense: 5′-UCUGAUCGCACGUAUGAUC-3′ ; AhR siRNA (SASI_Hs02_00332181, Sigma). The following shRNAs were obtained from the National RNAi Core Facility at Academic Sinica: control shRNA, 5′-TACAACAGCCACAACGTCTAT-3′; AhR shRNA (1) (TRCN0000021255), AhR shRNA (2) (TRCN00000245285), NF-κB shRNA (1) (TRCN0000006518), and NF-κB shRNA (2) (TRCN0000006520). Cells were transfected with siRNA (10 nM) or shRNA (2 μg) using LT1 transfection reagent.

### Immunoblot analysis

Whole cell extracts were prepared in RIPA lysis buffer (Millipore, **Te**mecula, CA, USA) containing 1× protease inhibitor cocktail (Thermo Scientific, Waltham, MA, USA). Protein concentrations were determined using a BCA protein assay kit (Thermo Scientific, Rockford, IL, USA). Equal amounts of protein (50 μg protein) were resolved by sodium dodecyl sulfate-polyacaryamide gel electrophoresis (SDS-PAGE), transferred onto a polyvinylidene difluoride membrane, and blocked with 5% nonfat dry milk for 1 hour at room temperature. After blocking, the membrane was incubated overnight with primary antibodies at 4°C and washed three times with PBST. The horseradish peroxidase conjugated secondary antibodies (Santa Cruz Biotechnology) were incubated for 1 hour at room temperature. The blots were washed three times with PBST and then visualized with an enhanced chemiluminescence kit (Thermo Scientific, Rockford, IL, USA). The primary antibodies were as follows: AhR, Gα_q/11_, G_β_, PIP2, IP3R, and PI3K from Santa Cruz Biotechnology (1:100); p44/42 MAPK (Erk1/2), Akt, p-Akt (ser473), NFκB, LaminA/C, Histion H3 and α-tubulin from Cell Signaling Technology (1:1000); COX-2 from Abcam (1:1000); β-actin from Sigma (1:1000).

### Immunoprecipitation

After preclearing for 30 minutes with protein G agarose (Millipore), antibodies specific for Gα_q/11_, G_β_, or PI3K (1:100; Santa Cruz Biotechnology) or IgG (2 μg, Sigma-Aldrich Co, St. Louis, MO, USA) were added before overnight incubation at 4°C, followed by precipitation for 2 hours with protein G agarose. The beads were washed three times with RIPA lysis buffer, boiled in sample buffer, and the protein were resolved by 8% SDS-PAGE before performing immunoblot analysis of the indicated proteins.

### Transwell migration and invasion assays

Cell migration assays were performed in 24-well inserts (8-μm pore size; BD Biosciences, Franklin Lakes, NJ, USA) and cell invasion assays were performed in 24-well Matrigel™ Invasion inserts (8-μm pore size; BD Biosciences, Franklin Lakes, NJ, USA). Cells (1 × 10^4^) in serum-free DMEM were seeded in the upper chamber of the insert and DMEM containing 10% fetal bovine serum was added to the lower chamber and followed by incubation for 1 day (for migration) or 2 days (for invasion). The medium and cells were then removed from the top chamber using cotton swabs and PBS. The cells were fixed with 4% paraformaldehyde for 30 minutes, stained with a 0.5% crystal violet solution for 2 hours, and counted under a microscopy.

### Measurement of vascular endothelial growth factor

Huh7 cells were grown in 12-well plates and treated with BBP for 1 day. After treatment, the cells were incubated in fresh medium for 1 day. The media were then collected and centrifuged at 1,000 rpm for 5 minutes to remove cell debris. Vascular endothelial growth factor (VEGF) levels in the conditioned medium were measured with an enzymed-linked immunosorbent assay (ELISA) kit (R&D System, Minneapolis, MN, USA).

### Angiogenesis tube formation assay

HUVEC (2 × 10^4^ cells/mL) were seeded in a 48-well plate pre-coated with Matrigel (BD Biosciences, San Jose, CA, USA). After Huh7 cell-conditioned medium was added to a final volume of 20%, the cells were cultured for 16 hours, stained with calcium-AM (Invitrogen), and visualized under a fluorescence microscope (Olympus). Total tube lengths were measured by MetaMorph software (Leica)

### Animals

Male 6-week-old nude mice (BALB/cA-nu nu/nu) were purchased from the National Science Council Animal Center (Taiwan). All animal experiments were performed according to a protocol approved by the Institutional Animal Care and Use Committee of Kaohsiung Medical University Hospital (IACUC Approval No: 101060).

### *In vivo*tumor xenograft experiments

Huh7 cells were stably transfected with infrared fluorescent protein [20]. Briefly, 293 T cells were transfected with pCMV-ΔR8.91, pMD.G, and pLKO AS3-reporter gene using LT1 transfection reagent for 3 days, and the supernatant (lentivirus-containing medium) was collected the next day. Huh7 cells (2 × 10^5^) were seeded into six-well plates and incubated for 1 day. The lentivirus-containing medium (200 μL) was mixed with 800 μL of DMEM containing 8 μg/mL polybrene and added to each well, and the cells were incubated for 1 day. A stable clone was selected by puromycin treatment (2 μg/mL) for 14 days. The cells were incubated with 25 μM biliverdin overnight and then purified Huh7-IFP cells by flow cytometry. The hepatocellular carcinoma model of direct intrahepatic injection was performed according to a previous study [21] with some modifications. After a small incision was made in nude mice to access the liver, Huh7-IFP cells (1 × 10^6^) suspended in PBS were slowly injected into the upper left lobe of the liver using a 28-gauge needle. A transparent bleb of cells was formed through the liver capsule after injection. To prevent bleeding, a small piece of sterile gauze was placed, and light pressure was applied on the injection site. After implantation, the mice were placed on a heating pad or below a heating lamp until fully active. The mice were randomly divided into two groups (vehicle or BBP treatment), 18 mice of each. After 3 days, BBP (500 mg/kg) was administered by intraperitoneal (i.p.) injection every 2 days. Previous studies have reported that administration of BBP by i.p. at a dose of 800 mg/kg for 24 weeks results in no significant toxic effects [11, 22], which is a higher dose than that used in this study. Tumor growth was detected by injecting biliverdin (250 nM) into the tail vein 30 minutes before imaging. After 1 month, the mice were sacrificed and the organs (liver, lungs, kidneys, and spleen) were removed and viewed with an Ultra Sensitive Molecular Imaging System (Berthold Technology). The numbers of the organs (lung, kidneys, spleen) that expressed fluorescence, considered as metastasis positive organs were determined.

### Immunohistochemistry

Liver tissues were fixed with 4% paraformaldehyde, embedded in paraffin, and cut into 4-μm thick sections. The sections were deparaffinized in xylene and rehydrated with a graded series of ethanol/water solutions (100% and 95% ethanol) and then water washes. The sections were treated with 10 mM citrate buffer at 95°C to retrieve antigens and blocked with 5% bovine serum albumin. Primary antibodies against PI3K (1:100; Santa Cruz Biotechnology) and NF-κB (1:200; Cell Signaling) were applied to the sections at 4°C overnight, and then the sections were incubated with secondary antibodies and 3,3’-diaminobenzidine. Intensities of PI3K and NF-κB staining were analyzed by Tissue Quest software (Tissue Genomics)

### In vivo Matrigel™ -plug angiogenesis assay

Huh7 cells (3 × 10^6^) were suspended in 150 μL PBS, mixed with 50 μL Matrigel (BD Biosciences), and injected into the flanks of nude mice. BBP (1 μM) was added to the cell suspensions of the treatment groups. After 21 days, the Matrigel plugs were removed. Hemoglobin levels were determined by Drabkin reagent (Sigma), and protein concentrations were normalized to measure blood vessel formation.

### Statistical analysis

Statistical significance was established using the Student’s *t*-test. A *p*-value of < 0.05 was considered statistically significant.

## Results

### BBP induced AhR expression

**Figure 1:**
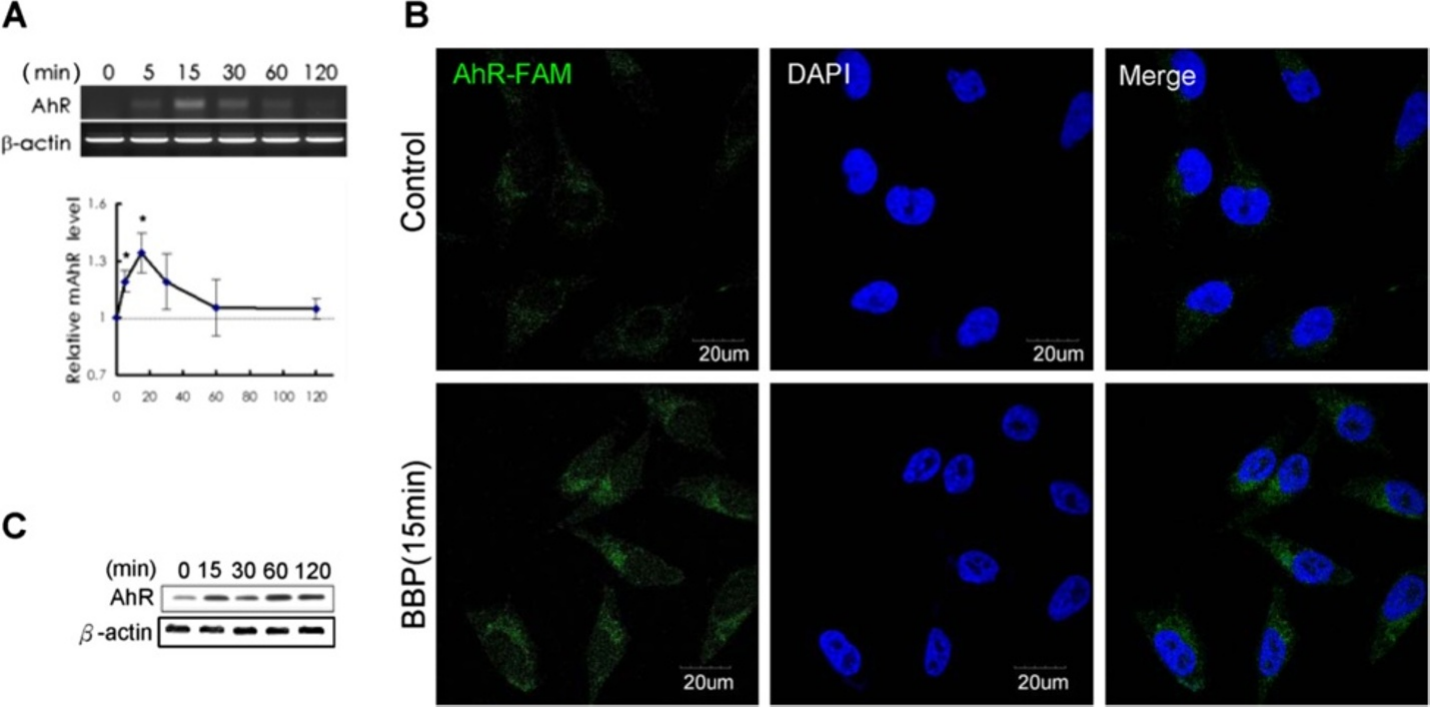
**BBP activates** ***AhR*** **mRNA and protein expression. (A)** Huh7 cells were treated with BBP (1 μM), and mRNA expression of AhR was analyzed by RT-PCR at the indicated time points. The agarose gel image is the expression of *AhR* mRNA. Each values in the graph was obtained by densitometry and is the mean of three independent experiments. Each value in the graph is the mean ± SD from three independent experiments. The asterisks indicate a significant difference expression relative to the level at 0 minute, as analyzed by Student’s *t*-test (*p* < 0.05). **(B)** Huh7 cells were treated with BBP (1 μM) or DMSO as the control group for 15 minutes, and then *AhR* mRNA was stained by fluorescence (carboxyfluorescein, FAM) *in situ* hybridization. Imaging was performed by confocal microscopy. **(C)** Huh7 cells were treated with BBP (1 μM), and then AhR levels were analyzed by immunoblotting at the indicated time.

The effect of BBP on *AhR* mRNA expression was examined by RT-PCR. BBP transiently increased *AhR* mRNA expression until it reached its highest level at 15 minutes after treatment (Figure 1A). Next, we examined the RNA level using a specific *AhR* mRNA probe. As a result, *AhR* mRNA expression was increased at 15 minutes after BBP teratment, which was comparable with the RT-PCR results (Figure 1B). Immunoblot analysis of the effect of BBP on AhR expression showed that BBP stimulated AhR expression in a time-dependent manner (Figure 1C).

### BBP activates AhR at the cell membrane, which interacts with G proteins

**Figure 2:**
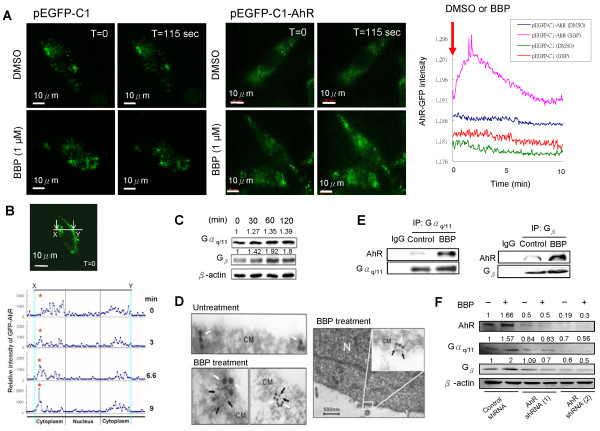
**BBP activates AhR at the cell membrane, which interacts with G proteins. (A)** Huh7 cells were transfected with pEGFP-C1-AhR or pEGFP-C1 as a plasmid control. Cells were stimulated by adding DMSO or BBP (1 μM) and then analyzed by real-time TIRF microscopy. Scale bars: 10 μm. The left panel shows Huh7 cells transfected with the pEGFP-C1 plasmid control treated with DMSO (upper) or BBP (lower). The middle panel shows Huh7 cells transfected with pEGFP-C1-AhR and then treated with DMSO (upper) or BBP (lower). The increased intensity of GFP fluorescence indicates AhR expression at the cell membrane, which was induced by BBP. The right panel shows the GFP intensity analyzed by Axio Vision Rel 4.8 software. **(B)** Huh7 cells were transfected with pEGFP-C1-AhR and then stimulated with BBP (1 μM) before analyzed by real-time confocal microscopy (upper panel), Scale bars: 10 μm. The GFP intensity was analyzed by FV10-ASW 2.1 software (Olympus) (lower panel). **(C)** Expression of Gα_q/11_ and G_β_ proteins after BBP treatment for the indicated time was detected by immunoblotting. β-actin was used as an internal control. **(D)** Interaction of AhR with Gα_q/11_ at the cell membrane after treatment with BBP (1 μM) was imaged by double immunogold electron microscopy. Black arrows indicate Gα_q/11_, and the white arrows indicate AhR. The localization of G protein and AhR protein are shown. (upper left panel) shows the untreatment group and (Lower left, Right left panel) indicated BBP treatment groups. Scale bars: 500 nm. CM, cell membrane; N, nucleus. **(E)** Huh7 cells after 30 minutes of treatment with BBP (1 μM) or DMSO as the control. The interaction of AhR with Gα_q/11_ and G_β_ was detected by immunoprecipitation (IP) followed by immunoblot analysis. The IgG were used cell lysates mixed with control and BBP treatment group. Normal rabbit IgG were used as negative control. **(F)** Huh7 cells were transfected with two different AhR shRNAs as described the Methods or a control shRNA. After treatment with or without BBP (1 μM), AhR, Gα_q/11_, and G_β_ levels were measured by immunoblotting. β-actin was used as an internal control.

To investigate whether AhR can be activated at the cell membrane by BBP, Huh7 cells were transfected with pEGFP-C1 as a plasmid control or pEGFP-C1-AhR treated with DMSO as a viechle control or BBP, and then analyzed by TIRF microscopy. AhR-GFP expression peaked at 2 minutes after BBP treatment (Figure 2A). Analysis of AhR movement in Huh7 cells showed that AhR expression near the membrane increased in a time**-**dependent manner. The experiment was performed by confocal microscopy and analyzed by FlowView 3.0 (Olympus, Japan) (Figure 2B). Stimulation of both Gα_q/11_and G_β_ expression by BBP was analyzed by immunoblotting (Figure 2C). Double immunogold transmission electron microscopy and immunoprecipitation (Figure 2D, 2E) further showed an interaction between AhR and G protein**s**. The action of AhR was notably nongenomic. To investigate whether the G-protein signaling induced by BBP was AhR dependent, we knocked down AhR using an AhR shRNA. The results showed that BBP-induced Gα_q/11_ and G_β_ expression was suppressed by transfection of the AhR shRNA (Figure 2F).

### Initial activation of COX-2 by AhR/Gα_q/11_ signaling

**Figure 3:**
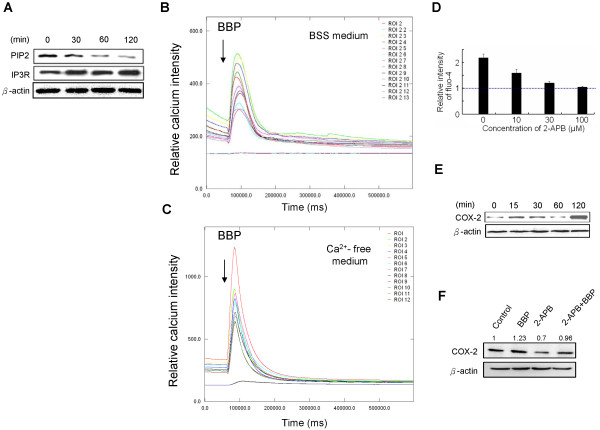
**AhR triggers Gα**_**q/11**_ **/calcium/COX-2 signaling. (A)** BBP induced a reduction in the level of PIP2 and activation of IP3R via Gα_q/11_. After Huh7 cells treated with BBP (1 μM), protein levels were analyzed by immunoblotting at the indicated time points. **(B)** Real-time imaging of calcium was performed by Cell-R microscopy. The experiment was performed in BSS medium. **(C)** Elevated intracellular calcium was induced by BBP treatment. The experiment was performed with a calcium free medium. The arrows indicate the time points of BBP (1 μM) addition (1 minute after the experiment started). The fluorescence intensity (Y-axis) indicates the relative calcium levels. **(D)** Huh7 cells in calcium-free medium were pretreated with various concentrations of 2-APB for 30 minutes before stimulation with BBP (1 μM). Internal calcium release was inhibited by 2-APB in a dose-dependent manner. The relative intensity of fluo-4 indicates the calcium levels: peak/baseline ratio of fluorescence intensity. Calcium-free medium was used for each experimental interval. Each value in the graph is the mean ± SD of six replicate using at least ten cells. **(E)** COX-2 expression after BBP treatment was measured by immunoblotting. Huh7 cells were treated with BBP (1 μM) for the indicated times before harvesting the cells. β-actin was used as an internal control. **(F)** Huh7 cells were pretreated with 2-APB (20 μM) for 2 hours and followed by treated with BBP (1 μM). Control groups were treated with DMSO. COX-2 levels were suppressed by 2-APB pretreatment.

Downstream signaling triggered by Gα_q/11_ was measured by immunoblot analysis of Gα_q/11_, PIP2 and IP3R levels. A decrease in PIP2 and an increase in IP3R indicated cleavage of PIP2 to IP3, followed by activation of IP3R (Figure 3A) [23]. The calcium response to BBP was then analyzed with a live-cell calcium imaging system that showed a sharp signal immediately after BBP addition (Figure 3B). To determine whether the calcium was derived from external or internal stores, calcium-free medium was used in a second round of experiments. As a result, calcium release was quickly stimulated by addition of BBP, presumably from internal stores (Figure 3C). The results were then confirmed using 2-APB, an IP3R inhibitor. 2-APB inhibited the internal release of calcium in a dose-dependent manner (Figure 3D). Expression of COX-2 was activated by BBP and inhibited by 2-APB (Figure 3E, 3F). These results suggest that BBP promotes COX-2 expression via AhR/Gα_q/11_/calcium signaling.

### Effect of BBP on cell migration and invasion through AhR/G_β_/PI3K/Akt/NF-κB signaling

**Figure 4:**
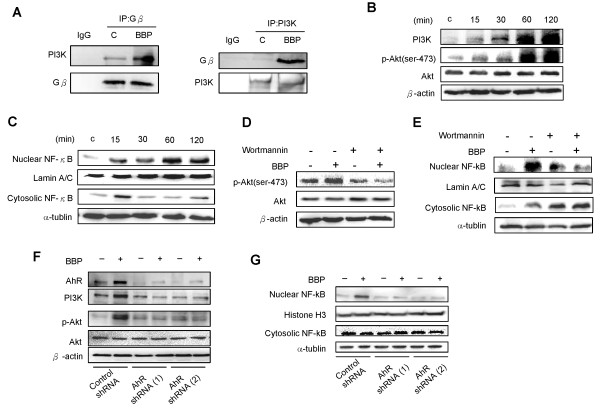
**AhR triggers G**_**β**_ **/PI3K/Akt/NF-κB signaling. (A)** The PI3K and G_β_ protein interaction was assessed by immunoprecipitation. Huh7 cells were immunoprecipitated with antibodies against G_β_ or PI3K. Normal IgG was used as a negative control. **(B)** Cells were treated with BBP (1 μM) for various durations, and then PI3K and phosphorylation levels of Akt were determined by immunoblotting. **(C)** Nuclear and cytoplasmic fractions of NF-κB were detected by immunoblotting. Lamin A/C and α-tubulin were used as internal markers for nuclear and cytoplasmic proteins, respectively. Huh7 cells were pretreated with wortmannin (100 nM) and then treated with BBP (1 μM) for 2 hours. **(D)** The Akt phosphorylation levels were measured and β-actin was used as an internal control. **(E)** The nuclear and cytoplasmic fractions of NF-κB were analyzed by immunoblotting. Huh7 cells were transfected with two different shRNA and treated with BBP (1 μM) for 2 hours. **(F)** PI3K, p-Akt, Akt levels were measured by immunoblotting. β-actin was used as an internal control. **(G)** The nuclear and cytoplasmic fractions of NF-κB were analyzed by immunoblotting. Histone H3 and α-tubulin were used as internal markers for nuclear and cytoplasmic proteins, respectively.

**Figure 5:**
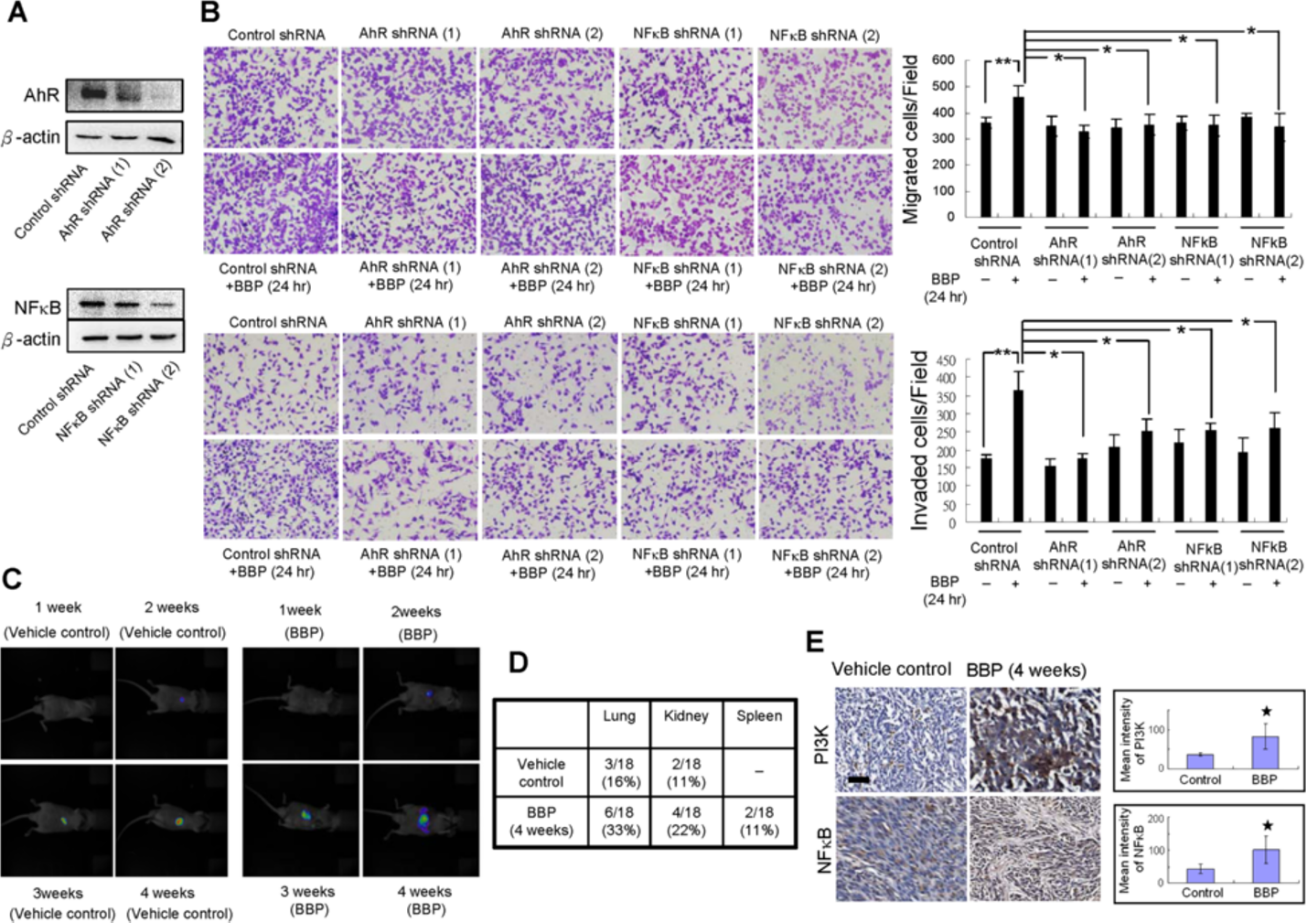
**Effects of BBP on cell migration and invasion** ***in vitro*** **and metastasis** ***in vivo.*** **(A)** Huh7 cells were transfected with two different AhR (top) and NF-κB (bottom) shRNA as described in the Methods, and then protein levels were detected by immunoblotting. **(B)** Transwell migration and invasion assays. Huh7 cells transfected with control, AhR, or NF-κB shRNAs were seeded onto inserts, treated with or without BBP, and allowed to migrate for 24 (migration) or 48 (invasion) hours. The numbers of migrating and invading cells were counted under a microscope. Scale bars: 100 μm. **(C)** Tumor growth of intrahepatically injected Huh7-IFP cells in vehicle control and BBP treatment groups for the indicated times of was detected by non-invasive imaging. **(D)** Numbers of other organs with metastasis of vehicle control or BBP treatment group (n = 18 for each group) after 4 weeks. **(E)** Immunohistochemical staining for PI3K and NF-κB in the tumor region of the mouse liver after treatment of BBP for 4 weeks. Magnificantion, 40x. The intensity of PI3K and NF-κB staining was analyzed by Tissue Quest software. Data shown in panels B and E are representative of three independent experiments. Each value is the mean ± SD of three independent experiments. The asterisks indicate a significant difference between vehicle treated group and BBP treated groups, as analyzed by Student’s *t*-test (*p < 0.05; **p < 0.01).

G_β_ protein activates PI3K by direct binding [24]. Immunoprecipitation was performed to examine the effect of BBP treatment on interactions between G_β_ protein and PI3K (Figure 4A). The binding of G_β_ protein to PI3K was increased after BBP treatment. To further study the downstream pathway triggered by G_β_ activation, we analyzed PI3K enhancement and Akt phosphorylation by immunoblotting. PI3K and Akt phosphorylation level were increased after BBP treatment (Figure 4B). We also found translocation of NF-κB into the nucleus (Figure 4C). Treatment with a PI3K inhibitor (wortmannin) reduced Akt phosphorylation (Figure 4D) and inhibited NF-κB translocation into the nucleus (Figure 4E). To further confirm whether PI3K/Akt/ NF-κB activation is specifically stimulated by BBP via AhR, we transfected Huh7 cells with two different AhR shRNAs. The increase of PI3K and p-Akt (Figure 4F), and translocation of NF-κB into the nucleus (Figure 4G) induced by BBP were inhibited by transfection of the shRNAs. PI3K/Akt/NF-κB enhances cell migration and invasion [25]. Thus, we further investigated the mechanisms of cell migration and invasion which are induced by BBP. Huh7 cells were transfected with two different AhR shRNAs, NF-κB shRNA, or control shRNA for 48 hours, followed by preparation of cell lysates. The protein levels of AhR and NF-κB markedly decreased after transfection with AhR and NF-κB shRNAs compared with those after transfection with control shRNA (Figure 5A). To further study the effects of BBP on Huh7 cells migration and invasion, transwell migration and invasion assays were performed. AhR and NF-κB shRNAs inhibited cell migration and invasion induced by BBP (Figure 5B). These results suggest that BBP promotes cell migration and invasion by activation of AhR/G_β_/PI3K/Akt/NF-κB signaling.

### BBP promotes *in vivo*metastasis

The mouse intrahepatic injection model was established to examine tumor metastasis [26]. Huh7-IFP cells (1 × 10^6^) were suspended in 30 μL PBS and injected into the mouse liver (At 3 days after of implantation, 500 mg/kg BBP was administered by i.p. injection every 2 days. Weekly imaging of the mice confirmed that the tumor sizes increased over time (Figure 5C). The mice were sacrificed after 1 month to compare tumor metastasis between control and BBP treatment groups. The metastasis rates in the lungs, kidneys, and spleen were higher in the BBP treatment group than those in the control group (Figure 5D), suggesting that BBP promotes metastasis. Immunohistochemistry showed that liver PI3K and NF-κB levels were significantly higher in the treatment group than those in the control group (Figure 5E).

### BBP promotes angiogenesis in vitro and in vivo

**Figure 6:**
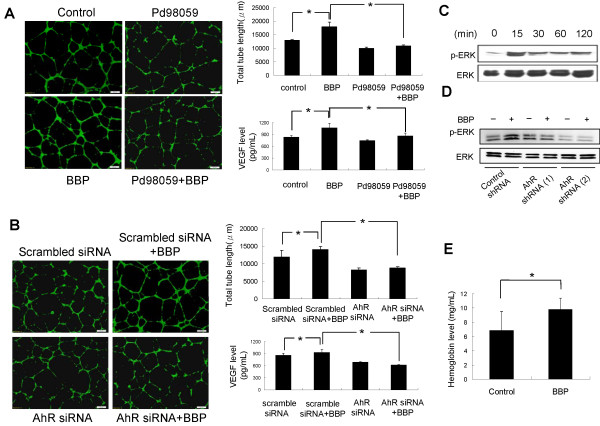
**Effects of BBP on angiogenesis** ***in vitro*** **and** ***in vivo.*** Effect of Huh7 cell-conditioned medium on HUVEC tube formation. Huh7 cells were pretreated with or without Pd98059 **(A)** or transfected with scrambled siRNA or AhR siRNA **(B)** followed by BBP treatment, and the conditioned medium was then collected from each culture dish. HUVEC were seeded in BioCoated angiogenesis plates, treated with 20% Huh7-conditioned medium, and incubated for 16 hours. HUVEC were imaged after staining with Calcein-AM. Total tube lengths were analyzed by MetaMorph software. Scale bars: 200 μm. VEGF levels in the conditioned media were measured by ELISA. **(C)** Huh7 cells were treated with BBP for the indicated times. p-ERK and ERK levels were measured by immunoblotting. β-actin used as an internal control. **(D)** Huh7 cells were transfected with two different Huh7 shRNAs and then treated with BBP (1 μM) for 15 minutes. p-ERK, ERK levels were evaluated by immunoblotting. **(E)** Huh7 cells were mixed with Matrigel with or without BBP (1 μM), injected into the flanks of nude mice, and allowed to grow for 21 days. Hemoglobin levels in Matrigel plugs, which indicated blood vessel formation, were measured by Drabkin reagent kit 525. Each value is the mean ± SD of three independent experiments. The asterisks indicate a significant difference between control and test groups, as analyzed by Student’s *t*-test (*p < 0.05; **p < 0.01).

To examine the effect of BBP on angiogenesis, the conditioned medium of Huh7 cells that had been treated with BBP was examined for its ability to induce the formation of capillary-like structures by HUVEC (Figure 6A). The levels of VEGF, which promotes angiogenesis [27], were also measured in the conditioned medium (Figure 6A). To explore the associated mechanisms, Huh7 cells were treated with the ERK inhibitor Pd98059 (Figure 6A) or transfected with AhR siRNA (Figure 6B) before collection of the medium. Analyses of the media showed that both Pd98059 and AhR siRNA inhibited tube formation of HUVEC and reduced VEGF induction after BBP treatment. Moreover, we evaluated the phosphorylation levels of ERK (Figure 6C). To further confirm whether activation of ERK was AhR dependent, we trasfected Huh7 cells with two different AhR shRNAs and the results showed inhibition of the phosphorylation levels of ERK induced by BBP (Figure 6D). The angiogenic effects of BBP were then assessed in an *in vivo* Matrigel plug angiogenesis assay model. Briefly, Huh7 cells were mixed with Matrigel and injected into the flanks of nude mice. After 3 weeks, the mice were sacrificed, and hemoglobin levels in the plug were measured. Hemoglobin levels in the Matrigel plug treated with BBP were significantly higher than those in the control group (Figure 6E).

## Discussion

In this study, we provide evidence that phthalate promotes hepatocellular carcinoma progression through a nongenomic AhR pathway. Rodent studies of the carcinogenesis of phthalate have yielded substantial data [5]. Some human epidemiological studies have also shown a cancer risk associated with phthalate exposure, including respiratory cancer [28], pancreatic cancer [29], and breast cancer [30]. However, the mechanisms of carcinogenesis have been rarely explored for phthalate. To further investigate such mechanisms, we treated hepatocellular carcinoma cell lines, Huh7, HepG2 and PLC cells with BBP (1 μM). All cell lines showed the activation of AhR after treatment (Additional file 1: Figure S1). We then tested the functions of cells treated with BBP including migration, invasion and angiogenesis. We found that BBP induced migration of Huh7 and PLC cell lines. In addition, BBP induced invasion and angiogenesis of Huh7 cells (Additional file 2: Figure S2). These results may be due to the higher constitutional level of AhR in Huh7 than in that in PLC cells (Additional file 2: Figure S2D). HepG2 cells are not appropriate for further animal studies for non-tumorigenic properties in immunosuppressed mice [31]. Therefore, we further investigated the mechanism induced by BBP in Huh7 cells. We used Huh7 cells in the *in vivo* study, and clarified the effect of BBP induced metastasis and angiogenesis *in vivo*.

Our current study showed that BBP treatment induces a nongenomic function through AhR and further clarified that AhR translocates to the membrane and subsequently activates G-protein signaling. Our investigation is consistent with a previous report in the nongenomic AhR mechanism induced by TCDD, showing that TCDD rapidly increases calcium concentrations and induces COX-2 expression in U937 macrophages, mouse MMDD1 macula densa cells, and MCF10 cells [14, 17]. We believe that the nongenomic action explains why cells elict fast inflammatory responses to the environmental pollutant TCDD and the endocrine disrupting agent, phthalate. After BBP treatment, the *AhR* mRNA levels were unregulated. Certain molecular such as NF-κB, interleukin (IL)-27 and IL-6 were investigated that can regulate AhR expression [32–34]. However, the mechanism of BBP induced AhR mRNA upregulation has remained unclear. Subsequently the nongenomic action triggers the cell signal pathways and affects the cell function. A previous study showed that by interacting with AhR-interacting protein, G_α13_ destabilized AhR via the ubiquitin-proteasome pathway [18]. Polycyclic aromatic hydrocarbons (PAH), which is the AhR ligand, induceds expression of AhR target genes through directed bind to G protein coupled receptor and activates G protein/ cAMP/ 1,4,5-trisphosphate (IP3) pathway, which results in calcium induction [35]. The current study further confirmed that AhR positively regulates G proteins, including Gα_q/11_ and G_β_ through nongenomic mechnissams. To our knowledge, the present study is the first investigation to elicit that phthalate activates AhR through a non-genomic mechanism that involving G protein signaling. Importantly, we provide evidence that the tumor progression, including migration, invasion and angiogenesis were regulated by this phthalate-induced signaling pathways.

Immortalized fibroblasts from *Ahr*^**−/−**^ mice demonstrate a lower tumorigenic potential because of diminished cell motility and responses to angiogenic factors [36]. Overexpression of AhR promotes the development of malignant cellular phenotypes and also increases their cell proliferation rate, motility, migration, and confers the ability to invade Matrigel in immortalized normal human mammary epithelial cells [37]. Activation of AhR is functionally connected to a signaling cascade that dramatically alters plasticity and increases motility in MCF-7 cells [38].

Angiogenesis contributes to tumor metastasis [39]. It has been reported AhR regulates both *in vitro* and *in vivo* angiogenesis. AhR^−/−^ endothelial cells fail to form tube-like structures, and impaired angiogenesis limits tumor xenograft growth in AhR null mice [40]. The current study showed that BBP promoted angiogenesis through nongenomic AhR mechanisms, and VEGF expression was increased by ERK1/2 phosphorylation. This finding is consistent with an earlier study showing that an ER-dependent mechanism involving activation of MEK, p38 kinase, and PI3K pathways by xenoestrogens up-regulates VEGF expression in breast cancer cells [41]. However, our current study is the first to show that BBP increases both *in vitro* and *in vivo* angiogenesis of liver cancer.

Previous reports have revealed that phthalates promote cancer cell migration, invasion and epithelial-mesenchymal transition, which may explain the cancer progression observed in both ER-dependent and AhR-dependent pathways [10, 42–44]. Several reports have shown that AhR regulates cell migration, invasion and plasticity, all of which contribute to tumor progression [10, 36, 45–47]. Our study showed that BBP promoted cell migration and invasion through the AhR/G_β_/PI3K/Akt/NF-κB pathway in hepatocellular carcinoma cells. These indicate phthalate stimulates various cell signaling pathways of cancer cell migration and invasion. Our data showed metastasis can be promoted by phthatlate *in vitro* and in an animal model *in vivo*. This is a phenomenon need to be addressed as metastasis is the key for the prognosis of cancer treatment.

Overexpression of COX-2 occurs in many pre-malignant, malignant, and metastatic cancers, including hepatocellular carcinoma. COX-2 regulates carcinogenesis by regulating angiogenesis [48], suppressing the immune response, and by inhibiting apoptosis, and tumor cell invasion, and metastasis [49]. Our data suggest that BBP may contribute to angiogenesis, tumor cell invasion and metastasis by increasing COX-2 expression.

## Conclusions

**Figure 7:**
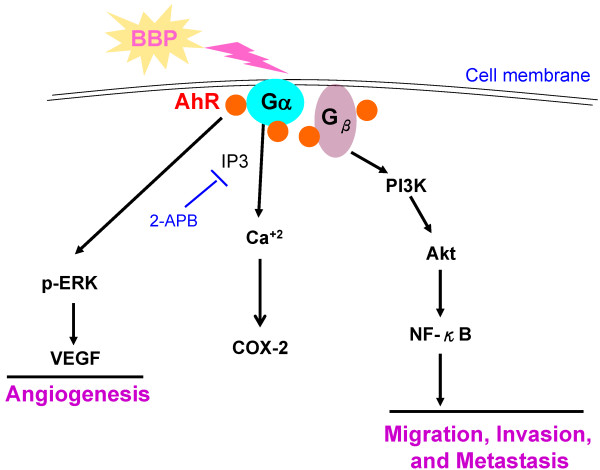
**Proposed signaling pathway of Huh7 cells treated with BBP (see the text for details).**

Taking these results together, we propose a signaling pathway for BBP stimulated hepatocellular carcinoma progression (Figure 7). BBP induces membrane translocation of AhR and the initial activation of COX-2 by AhR/Gα_q/11_ signaling. In addition, BBP promotes angiogenesis via the AhR/ERK/VEGF pathway and cell migration and invasion through AhR/G_β_/PI3K/Akt/NF-κB signaling.

In conclusion, we revealed a nongenomic AhR mechanism that may account for the modulated progression of liver cancer after phthalate exposure. Our results imply that BBP has a promoting impact on hepatocellular carcinoma, and that phthalate should be avoided in theses patients.

## Electronic supplementary material

Additional file 1: Figure S1: Effects of BBP on AhR expression of hepatocellular carcinoma cell lines. Huh7, HepG2, and PLC cells were treated with BBP (1 μM) for 24 hours and AhR protein levels were then analyzed by immunoblotting. β-actin was used as an internal control. (TIFF 512 KB)

Additional file 2: Figure S2: Effects of BBP on angiogenesis, migration and invasion. (A) Effect of conditioned media from Huh7 and PLC cells on HUVEC tube formation. After Huh7 and PLC cells were treated with BBP (1 μM) for 1 day, the culture medium was changed to the fresh medium and the cells were cultured for 1 day. Conditioned medium was collected from each culture dish. HUVEC were treated with 20% conditioned medium and incubated for 16 hours. HUVEC were imaged after staining with Calcein-AM (top). Total tube lengths were determined by MetaMorph software (bottom). Scale bar: 200 μm. The asterisks indicates a significant difference between control and test groups, as analyzed by Student’s t-test (*p < 0.05). (B) Transwell migration assay. (C) Transwell invasion assay. Huh7 and PLC cells were seeded on inserts and treated with BBP (1 μM). Scale bars: 100 μm. (D) Huh7 and PLC cells were harvested to prepare whole cell lysates for AhR protein levels were measured by immunoblotting, β-actin was used as an internal control. (TIFF 5 MB)

## Abbreviations

(BBP): Benzyl butyl phthalaye
(AhR): Aryl hydrocarbon receptor
(DEHP): Bis(2-ethylhexyl) phthalate
(TCDD): 2,3,7,8-tetrachlorodibenzo-p-dioxin
(2-APB): 2-aminoethoxydiphenyl borate
(DAPI): 6-diamidino-2-phenylindole
(PBS): Phosphate-buffered saline
(SSC): Saline-sodium citrate
(i.p): Intraperitoneal
(DRE): Dioxin response element.
