# Supplementary figures and images for: Benzyl butyl phthalate induces migration, invasion, and angiogenesis of Huh7 hepatocellular carcinoma cells through nongenomic AhR/G-protein signaling
Source: BMC Cancer. 2014 Aug 1;14:556. doi: 10.1186/1471-2407-14-556 (PMC4131049; doi:10.1186/1471-2407-14-556)

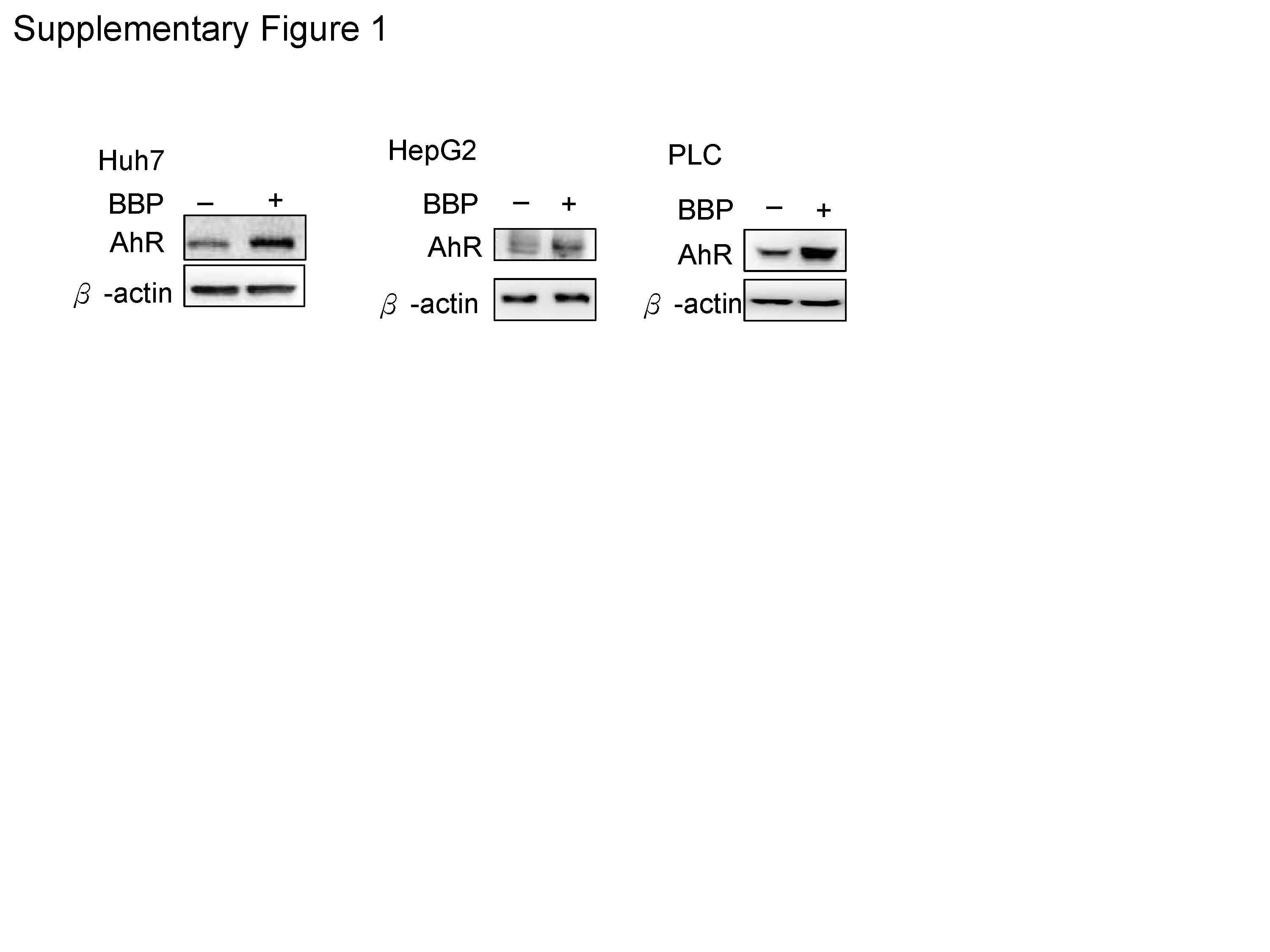

Supplement: Supplementary file 1 — Additional file 1: Figure S1: Effects of BBP on AhR expression of hepatocellular carcinoma cell lines. Huh7, HepG2, and PLC cells were treated with BBP (1 μM) for 24 hours and AhR protein levels were then analyzed by immunoblotting. β-actin was used as an internal control. (TIFF 512 KB) [file 12885_2013_4746_MOESM1_ESM.tiff]

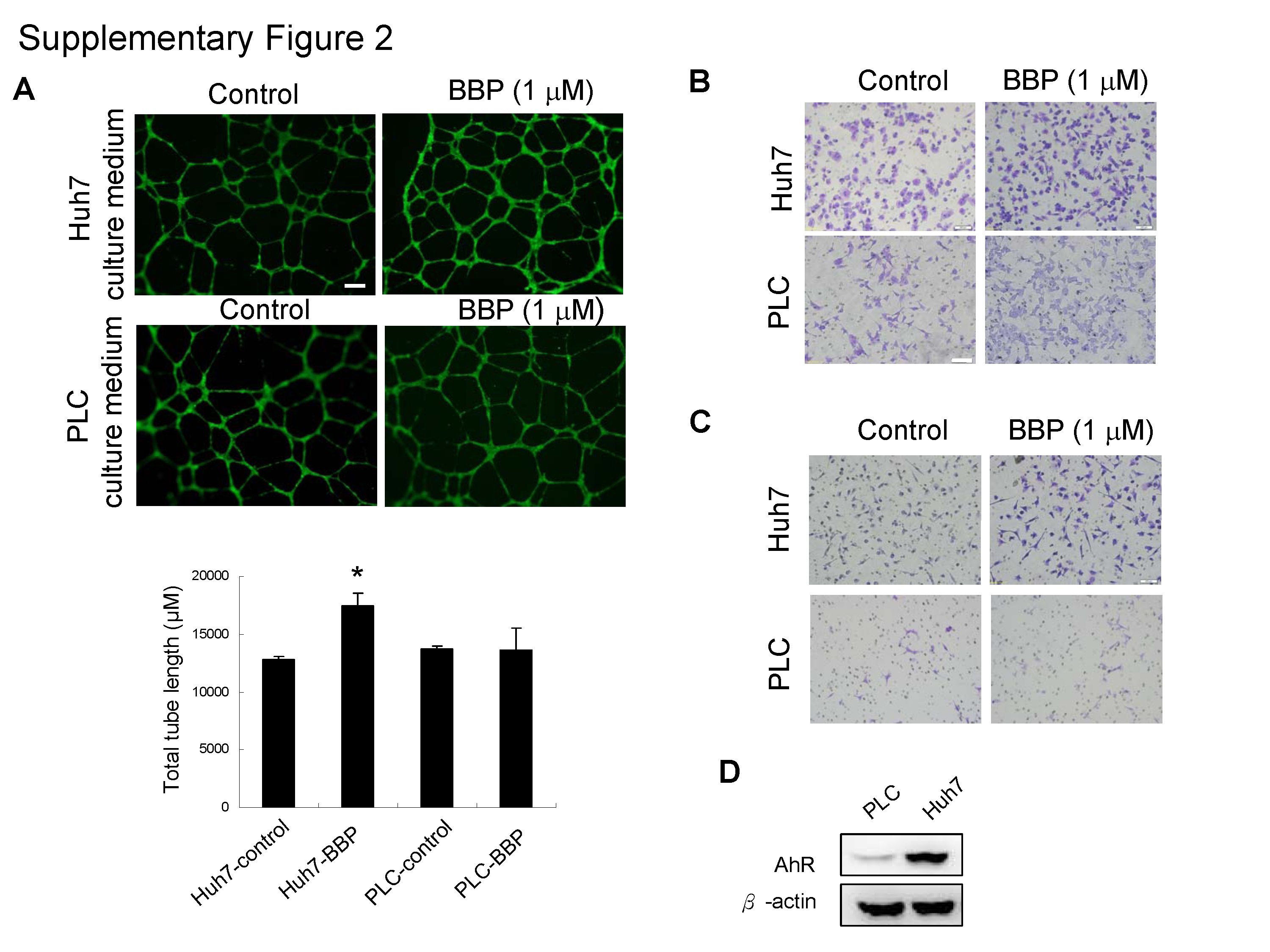

Supplement: Supplementary file 2 — Additional file 2: Figure S2: Effects of BBP on angiogenesis, migration and invasion. (A) Effect of conditioned media from Huh7 and PLC cells on HUVEC tube formation. After Huh7 and PLC cells were treated with BBP (1 μM) for 1 day, the culture medium was changed to the fresh medium and the cells were cultured for 1 day. Conditioned medium was collected from each culture dish. HUVEC were treated with 20% conditioned medium and incubated for 16 hours. HUVEC were imaged after staining with Calcein-AM (top). Total tube lengths were determined by MetaMorph software (bottom). Scale bar: 200 μm. The asterisks indicates a significant difference between control and test groups, as analyzed by Student’s t-test (*p < 0.05). (B) Transwell migration assay. (C) Transwell invasion assay. Huh7 and PLC cells were seeded on inserts and treated with BBP (1 μM). Scale bars: 100 μm. (D) Huh7 and PLC cells were harvested to prepare whole cell lysates for AhR protein levels were measured by immunoblotting, β-actin was used as an internal control. (TIFF 5 MB) [file 12885_2013_4746_MOESM2_ESM.tiff]
